# A nonlinear elastic description of cell preferential orientations over a stretched substrate

**DOI:** 10.1007/s10237-020-01406-4

**Published:** 2021-01-15

**Authors:** Giulio Lucci, Luigi Preziosi

**Affiliations:** 1 Department of Mathematical Sciences “G.L. Lagrange”, Politecnico di Torino, Corso Duca degli Abruzzi 24, 10129 Turin, Italy; 2 Department of Mathematics “G. Peano”, Università degli Studi di Torino, Via Carlo Alberto 10, 10123 Turin, Italy; 3 Department of Mathematical Sciences “G.L. Lagrange” Dipartimento di Eccellenza 2018-2022, Politecnico di Torino, Corso Duca degli Abruzzi 24, 10129 Turin, Italy

**Keywords:** Nonlinear elasticity, Orthotropic, Cell orientation, Cell stretching, Stress fibers, Cell mechanosensing, 74B20, 74L15, 92C10, 92C37

## Abstract

The active response of cells to mechanical cues due to their interaction with the environment has been of increasing interest, since it is involved in many physiological phenomena, pathologies, and in tissue engineering. In particular, several experiments have shown that, if a substrate with overlying cells is cyclically stretched, they will reorient to reach a well-defined angle between their major axis and the main stretching direction. Recent experimental findings, also supported by a linear elastic model, indicated that the minimization of an elastic energy might drive this reorientation process. Motivated by the fact that a similar behaviour is observed even for high strains, in this paper we address the problem in the framework of finite elasticity, in order to study the presence of nonlinear effects. We find that, for a very large class of constitutive orthotropic models and with very general assumptions, there is a single linear relationship between a parameter describing the biaxial deformation and $\cos ^2\theta _{\mathrm{eq}}$, where $\theta _{\mathrm{eq}}$ is the orientation angle of the cell, with the slope of the line depending on a specific combination of four parameters that characterize the nonlinear constitutive equation. We also study the effect of introducing a further dependence of the energy on the anisotropic invariants related to the square of the Cauchy–Green strain tensor. This leads to departures from the linear relationship mentioned above, that are again critically compared with experimental data.

## Introduction

From the biological point of view, it is nowadays recognized that, in addition to chemical cues, cells actively respond to mechanical stimuli exerted on them by the surrounding environment. Even though the precise subcellular mechanisms governing this interaction still need to be understood, there is consistent evidence that the cytoskeleton has a central importance in the mechanical response (Hayakawa et al. 2001; Neidlinger-Wilke et al. 2001; Wang 2000; Wang et al. 2001). In particular, contractile actin stress fibers display the ability to develop reactive mechanical forces and to reorganize their structure in response to external changes: this mechanosensing is mediated by focal adhesions (Chen et al. 2012), that is, protein assemblies through which the actin internal structure is linked to the extracellular matrix (ECM). Focal adhesions provide both an anchorage to the substrate and a signal transmission, coupling the cell with the outside environment by detection of mechanical changes: they convert these stimuli into biochemical pathways, inducing a remodelling of the cytoskeletal structure.

These mechanical interactions between the cell and its neighbourhood are shown to play a fundamental role in several physiological situations, like cell motion, cell differentiation (Vlaikou et al. 2017) and tumour and tissue development. For instance, it is well known that cancer development is related to the loss of contact inhibition from its beginning (see Chaplain et al. 2006 and references therein) and that ECM stiffness and cell tensile stress influence both their proliferation and death (Butcher et al. 2009; Kass et al. 2007; Kumar and Weaver 2009). Incorrect response to mechanical cues is also related to many other diseases such as atrial fibrillation, intimal hyperplasia, scleroderma, diabetic nephropathy, glomerulosclerosis, enphysema, pulmonary, and liver fibrosis (Ingber 2009).

Stem cell fate and stem cell culturing are influenced by the mechanical interactions with the environment as well (Butcher et al. 2009; Discher et al. 2009; Guilak et al. 2009; Sun et al. 2012; Tulloch et al. 2011; Yan et al. 2013). This is the reason why cell cultures in vitro and tissue-engineered samples tend to reproduce the correct environmental conditions that real cells would live in (Costa et al. 2012). Consequently, just to mention two relevant examples, muscle cells and cardiac cells are often grown on substrata that are cyclically stretched, in order to mimic respectively muscle contraction and heartbeat (Kim et al. 1999; Laflamme and Murry 2011; Yoon et al. 2017). Now, when subject to a periodic deformation, several types of cells (not only those that have a bipolar morphology, such as fibroblasts, myofibroblasts, myocytes, airway smooth muscle cells, but also endothelial cells (Moretti et al. 2004)) show a peculiar response that proves their subtle sensitivity to mechanical prompts: when laying on a substratum they tend to re-orient themselves until they reach a stable configuration, with a well-defined angle between their polarization axis (along which the stress fibers become mainly aligned) and the direction of stretching. This reorientation process involves the cytoskeleton, with disruption and rebuilding of actin stress fibers, and the cell body, that follows the cytoskeletal reorganization with some delay (Hayakawa et al. 2001).

In the last two decades, cell reorientation following mechanical deformations has been investigated in an attempt to gain insight into this complicated phenomenon (Faust et al. 2011; Hayakawa et al. 2001; Jungbauer et al. 2008; Moretti et al. 2004; Morioka et al. 2011; Neidlinger-Wilke et al. 2001, 2002; Wang et al. 2001, 2018). It is currently accepted that such a change in orientation is an active mechanism carried out by the cell (Wang et al. 2001, 2018) and that mechanical strain is the main driving force of this process. However, experimental settings show a broad variety of behaviours: the vast majority of them proved that cells head their major axis towards a direction oblique or nearly perpendicular to the direction of greater stretch (Chen et al. 2012; Hayakawa et al. 2001; Moretti et al. 2004; Neidlinger-Wilke et al. 2001; Wang et al. 2001), even though some authors reported different results (Bischofs and Schwarz 2003; Collinsworth et al. 2000). The final orientation angle reached by the cell is determined by several factors like the amplitude, the frequency (Chen et al. 2012; Jungbauer et al. 2008), the biaxiality ratio of the applied cyclic deformation (Livne et al. 2014), and the substrate stiffness is sometimes thought to have a role as well (Obbink-Huizer et al. 2014; Tondon and Kaunas 2014; Tondon et al. 2012). For sake of completeness, we also mention that in 3D cell behaviour is influenced by the fact that they are embedded in a network of ECM fibers all around. So, under stretch, adhering to the fibers they tend to align more with the stretching direction (Asano et al. 2018; Chen et al. 2018; Eastwood et al. 1998).

From the modelling point of view, some attempts to explain this phenomenon have been done using linear elasticity to describe cell and substrate behaviour. Many works focused on looking for the directions of minimal strains or minimal stress (De et al. 2007, 2008; Faust et al. 2011; Livne et al. 2014; Wang 2000; Wang et al. 1995) as a preferred orientation for cellular placement under uniaxial cyclic strain conditions. However, in a recent work Livne et al. (2014) studied the response of cells on a substrate subject to biaxial extensions, finding a linear relation between $\cos ^2\theta _{\mathrm{eq}}$, where $\theta _{\mathrm{eq}}$ is the angle formed by the principal strain direction and the most elongated axis of the cell, and $\varepsilon _{xx}/(\varepsilon _{xx}-\varepsilon _{yy})$, where $\varepsilon _{xx}$ and $\varepsilon _{yy}$ are the two infinitesimal principal strains of the biaxial test. The theoretical result correlated with the experimental data (Faust et al. 2011; Livne et al. 2014), suggesting that cells tend to minimize an elastic energy.

Nevertheless, it is worth observing that, in several experimental assays, mechanical tests were performed applying deformations for which using linear elasticity might be arguable, at least theoretically. As a matter of fact, for instance, in Livne et al. (2014) itself the maximum amplitude of the cyclic strain reached 24%, while in Faust et al. (2011) deformations up to 32% were applied to the specimen. In the latter work, the authors focused on the dependence of the orientation angle from the amplitude of the deformation, showing the existence of a stretch threshold necessary to induce a significant reorientation. Taken together, these results call for a study of the presence of nonlinear effects at high strains, in order to quantify their relevance in cellular mechanosensing and orientation dynamics. To our knowledge, only Lazopoulos and Pirentis (2007), Lazopoulos and Stamenović (2006) employed a finite elasticity framework to describe stress fibers reorganization in strained cells, although they considered only uniaxial substrate stretching and addressed the problem using a non-convex energy, giving an explanation based on the co-existence of phases.

The aim of this work is then to study the problem of cell reorientation in a nonlinear elasticity framework. The main goal is to understand why and to what extent the experimental results follow the same rule justified on the basis of linear elasticity also for large strains and are independent from the mechanical characteristics of the substratum and of the cells. We find that, considering the substratum with seeded cells as a finite-elastic orthotropic material, a very large class of elastic energies is minimized by a relationship that can be considered as the nonlinear generalization of the one found in Livne et al. (2014). Specifically, introducing the parameter $\Lambda :=\frac{\lambda _x-1}{\lambda _x-\lambda _y}$ (where $\lambda _x$ and $\lambda _y$ are the principal stretches and $\lambda _x > \lambda _y$ without loss of generality) that quantifies the biaxiality of the finite deformation, we put in evidence a linear relation between $\Lambda$ and $\cos ^2\theta _{\mathrm{eq}}$, where $\theta _{\mathrm{eq}}$ is the equilibrium angle of the cell with respect to the *x*-axis. The slope of such a straight line turns out to be a combination of the coefficients that multiply the anisotropic invariants based on the right Cauchy–Green strain tensor $\mathbb {C}$ and the polarization axis, which characterize the orthotropic constitutive model with quadratic dependency. The same relationship holds true for Fung-type materials as well, making the results valid for two energy forms often used in biomechanics.

Instead, a nonlinear dependence of the equilibrium from $\Lambda$ comes out visibly if the elastic energy also depends on the anisotropic invariants related to the square of the Cauchy–Green strain tensor, i.e. $\mathbb {C}^2$. In particular, we find that the presence of a non-vanishing coefficient in front of the term describing the response to stretch along the polarization axis gives rise to a departure from the linear behaviour that, for small values of the coefficient, is still compatible with experiments. Conversely, a non-vanishing coefficient in front of the term in charge of describing the response to stretch perpendicular to the polarization axis gives rise to results that look incompatible with experiments, suggesting this term is not present in the constitutive model. The dependence of the bifurcation point between the orientation perpendicular to the stretching direction and the oblique one is also examined for all cases, observing its disappearance above certain values of the energy coefficients.

In detail, the paper is organized as follows. In Sect. 2, we introduce the problem set-up and introduce the needed mathematical and mechanical background. In Sect. 3, we use the approach proposed in Saccomandi and Vianello (1997), Vianello (1996) to describe the stationary points for a general orthotropic constitutive equation. In Sect. 4, we focus on two very common types of energies employed in biological applications, namely a quadratic and Fung-type strain energy, showing that they behave identically as far as the stability problem is concerned. Afterwards, we calculate the specific equilibria and discuss their stability as a function of the involved material parameters. Section 5 is dedicated to the results in two significant cases, namely, in Sect. 5.1 we consider an energy which is independent of $\mathbb {C}^2$, showing the existence of the linear relationship mentioned above, while in Sect. 5.2 we consider the effects of including the invariants that involve the square of the Cauchy–Green strain tensor. The final Sect. 6 summarizes the results and discusses some possible directions for future research.

## Mechanical background and problem set-up

**Fig. 1:**
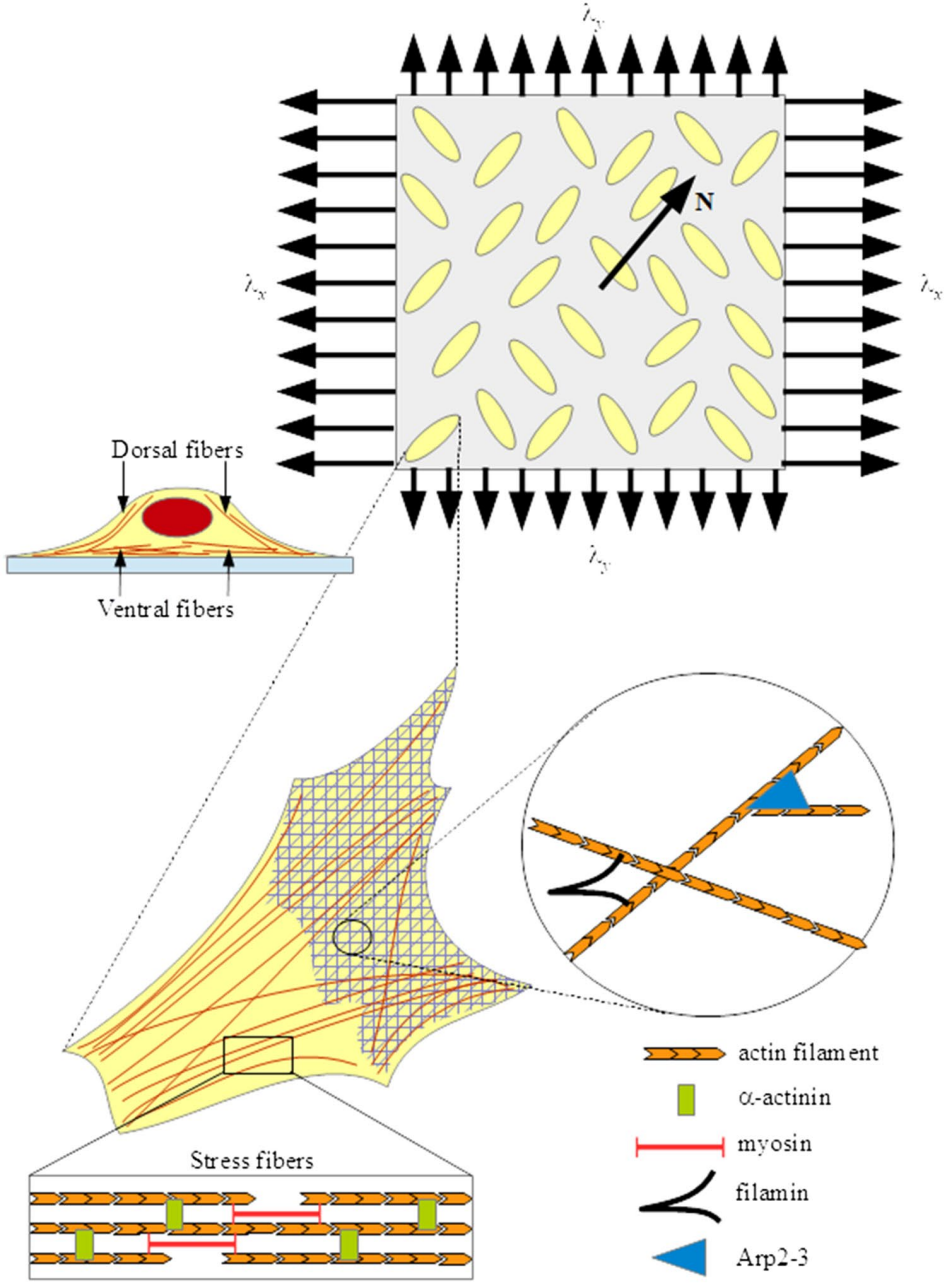
Sketch of experimental set-up and consequent inner structure of a typical cell

We consider a substratum provided with an ensemble of cells adherent to its surface in a subconfluent configuration, as in most experiments, which rules out the influence of cell-cell interaction. The system is then subject to a biaxial deformation, due to pulling and compression performed by an external device. As a consequence of this mechanical stimulus, following the deformation of the substrate cells orient on average along a certain direction, which can be identified through a unit vector $\mathbf {N}$ in the first quadrant of a reference system with the axes along the directions of the biaxial deformation (see Fig. 1).

If we assume that the system composed by the substrate and the overlying cells behaves as an elastic continuum, the deformation will induce a storage of energy into the body that depends on the orientation $\mathbf {N}$. We want here to study how the elastic strain energy of the system subject to a biaxial stretch depends on the average direction of the stress fibers $\mathbf {N}$.

For this purpose, we consider a general elastic energy density ${\mathcal {U}}$ for an orthotropic material

1 $$\begin{aligned} {\mathcal {U}} = {\mathcal {U}}(\mathrm{I}_1, \mathrm{I}_2, \mathrm{I}_3, \mathrm{I}_4, \mathrm{I}_5, \mathrm{I}_6, \mathrm{I}_7, \mathrm{I}_8), \end{aligned}$$

depending on the deformation gradient $\mathbb {F}$ through the invariants of the right Cauchy–Green deformation tensor $\mathbb {C}= \mathbb {F}^T\mathbb {F}$

2 $$\begin{aligned} \begin{array}{lll} \mathrm{I}_1:= \mathrm{tr}\, \mathbb {C}\,,& \mathrm{I}_2:= \dfrac{1}{2}\left[ (\mathrm{tr}\, \mathbb {C})^2-\mathrm{tr}\, \mathbb {C}^2\right] \,,& \mathrm{I}_3:=\det \mathbb {C}\,,\\ \mathrm{I}_4 := \mathbf {N}\cdot \mathbb {C}\mathbf {N}= |\mathbb {F}\mathbf {N}|^2, & \mathrm{I}_5 := \mathbf {N}\cdot \mathbb {C}^2\mathbf {N}= |\mathbb {C}\mathbf {N}|^2, \\ \mathrm{I}_6 := \mathbf {N}_{\perp }\cdot \mathbb {C}\mathbf {N}_{\perp } = |\mathbb {F}\mathbf {N}_{\perp }|^2 ,& \mathrm{I}_7 := \mathbf {N}_{\perp }\cdot \mathbb {C}^2\mathbf {N}_{\perp } = |\mathbb {C}\mathbf {N}_{\perp }|^2, \\ \mathrm{I}_8 := \mathbf {N}_{\perp }\cdot \mathbb {C}\mathbf {N}= (\mathbb {F}\mathbf {N}_{\perp })\cdot \mathbb {F}\mathbf {N}\,, \end{array} \end{aligned}$$

where $\mathbf {N}_\perp$ is the direction perpendicular to $\mathbf {N}$ in the plane of the substratum, which is also the plane containing the principal stretch. Notice that in (1) one could also expect a dependence on $\mathbf {N}_{\perp }\cdot \mathbb {C}^2\mathbf {N}$, but it is omitted since it depends on the other invariants (see, for instance, Ogden 2003).

This kind of energy is commonly used to describe the mechanical behaviour of anisotropic materials that show two preferential directions (Holzapfel and Gasser 2001; Ogden 2003). For instance, it is employed for fiber-reinforced materials, in which there are, say, two orthogonal bundles of fibers that influence the mechanical response of the body (Melnik and Goriely 2013), or for blood vessel walls that present fiber bundles in different directions. In our case, the system displays a natural anisotropy due to the presence in the cells of aligned stress fibers (SF) and actin filament structures that are cross-linked by several types of proteins, like fascin, fimbrin, $\alpha$-actinin, filamin, ARP2-3 (Civelekoglu-Scholey et al. 2005; Lu et al. 2008; Xu et al. 2016) (see Fig. 1). Since SF are two to even ten times stiffer than the lateral actin network (Lu et al. 2008; Mathur et al. 2007), they induce bidirectional anisotropy in the mechanical response, justifying the general assumption (1) to consider the system as orthotropic.

We also observe that a cell does not have a real polarization given by a head and a tail (Wang et al. 1995). So, configurations with cells aligned along $\mathbf {N}$ and $-\mathbf {N}$ are geometrically indistinguishable and therefore energetically equivalent. A similar thing holds true if we replace $\mathbf {N}_\perp$ with $-\mathbf {N}_\perp$. This implies that the elastic energy should be the same under the related symmetry transformations. All the invariants mentioned in (2) achieve the same values under the symmetry transformations above, except for $\mathrm{I}_8$ that changes sign, i.e. given $\mathbb {C}$, $\mathrm{I}_8(-\mathbf {N},\mathbf {N}_\perp ,\mathbb {C}) = -\mathrm{I}_8(\mathbf {N},\mathbf {N}_\perp ,\mathbb {C})$ and $\mathrm{I}_8(\mathbf {N},-\mathbf {N}_\perp ,\mathbb {C}) = -\mathrm{I}_8(\mathbf {N},\mathbf {N}_\perp ,\mathbb {C})$. Therefore, to satisfy the symmetry requirements, $\mathcal {U}$ must be an even function of $\mathrm{I}_8$.

If we orient the *x*- and *y*-axes of the reference frame along the principal stretching directions, the right Cauchy–Green tensor is diagonal and its eigenvalues are the principal stretches, i.e.

$$\begin{aligned} \mathbb {C}=\begin{pmatrix} \lambda _x & 0 & 0\\ 0 & \lambda _y & 0\\ 0 &0 &\lambda _z\\ \end{pmatrix}\,, \end{aligned}$$

with $\lambda _x>\lambda _y$ and $\lambda _x>1$. Actually, in the case of equi-biaxial deformation $\lambda _x=\lambda _y$, cells do not show a preferential orientation (Wang et al. 2001). Once the deformation is fixed, our goal is to study which orientations for the stress fibers correspond to minima of the elastic energy.

## Stationary points by the coaxiality approach

The general problem of finding the critical points of an hyperelastic anisotropic strain energy for a body subject to a rotation and a deformation has been studied in Saccomandi and Vianello (1997), Vianello (1996). In particular, it has been shown that the critical points of the energy are achieved for those rotations that make the stress and strain tensors coaxial. Since two symmetric tensors are coaxial if and only if they commute (Vianello 1996), we have to find the rotations $\mathbb {Q}$ about the *z*-axis such that

3 $$\begin{aligned} \mathbb {S}^*\mathbb {C}^* = \mathbb {C}^*\mathbb {S}^*, \end{aligned}$$

where $\mathbb {C}^* = \mathbb {Q}\mathbb {C}\mathbb {Q}^T$ and $\mathbb {S}^* = \mathbb {S}(\mathbb {C}^*)$ is the second Piola-Kirchhoff stress tensor corresponding to the deformation $\mathbb {C}^*$. To explicitly write $\mathbb {S}^*$ in our case, we define the structural tensors

4 $$\begin{aligned} \mathbb {A}:= \mathbf {N}\otimes \mathbf {N}\, , \quad \mathbb {A}_\perp := \mathbf {N}_\perp \otimes \mathbf {N}_\perp \, , \quad \mathbb {D}:= \frac{1}{2}[\mathbf {N}\otimes \mathbf {N}_\perp + \mathbf {N}_\perp \otimes \mathbf {N}] \end{aligned}$$

and recall that

5 $$\begin{aligned} \begin{array}{lll} & \dfrac{\partial \mathrm{I}_1}{\partial \mathbb {C}}(\mathbb {C}) = \mathbb {I} \, ,\qquad\quad \qquad \quad \dfrac{\partial \mathrm{I}_2}{\partial \mathbb {C}}(\mathbb {C}) = \mathrm{I}_1(\mathbb {C})\mathbb {I} - \mathbb {C}\, , \\ \\ & \dfrac{\partial \mathrm{I}_3}{\partial \mathbb {C}}(\mathbb {C}) = \mathrm{I}_3(\mathbb {C})\mathbb {C}^{-1} \, ,  \qquad \quad \dfrac{\partial \mathrm{I}_4}{\partial \mathbb {C}}(\mathbf {N}, \mathbb {C}) = \mathbb {A}\, , \\ \\ & \dfrac{\partial \mathrm{I}_5}{\partial \mathbb {C}}(\mathbf {N}, \mathbb {C}) = \mathbb {A}\mathbb {C}+ \mathbb {C}\mathbb {A}\, , \quad\, \,\,\dfrac{\partial \mathrm{I}_6}{\partial \mathbb {C}}(\mathbf {N}_\perp , \mathbb {C}) = \mathbb {A}_\perp \, ,\\  \\ & \dfrac{\partial \mathrm{I}_7}{\partial \mathbb {C}}(\mathbf {N}_\perp , \mathbb {C}) = \mathbb {A}_\perp \mathbb {C}+ \mathbb {C}\mathbb {A}_\perp \, , \dfrac{\partial \mathrm{I}_8}{\partial \mathbb {C}}(\mathbf {N}, \mathbf {N}_\perp , \mathbb {C}) = \mathbb {D}\, . \end{array} \end{aligned}$$

Therefore, the second Piola–Kirchhoff stress tensor reads

6 $$\begin{aligned} \mathbb {S}^* &=2\frac{\partial \mathcal {U}}{\partial \mathbb {C}}(\mathbb {C}^*) = 2\sum _{p = 1}^8 \, \frac{\partial \mathcal {U}}{\partial \mathrm{I}_p} \frac{\partial \mathrm{I}_p}{\partial \mathbb {C}}(\mathbb {C}^*)  \\ &=2[s_1 \mathbb {I} + s_2(\mathrm{I}_1(\mathbb {C}^*)\mathbb {I}-\mathbb {C}^*) + s_3 \mathrm{I}_3(\mathbb {C}^*)(\mathbb {C}^*)^{-1} + s_4 \mathbb {A}+ s_5(\mathbb {A}\mathbb {C}^* + \mathbb {C}^*\mathbb {A}) + s_6 \mathbb {A}_\perp \\&+ s_7(\mathbb {A}_\perp \mathbb {C}^* + \mathbb {C}^*\mathbb {A}_\perp ) + s_8 \mathbb {D}], \end{aligned}$$

where we have defined

$$\begin{aligned} s_p := \frac{\partial \mathcal {U}}{\partial \mathrm{I}_p}(\mathrm{I}_1(\mathbb {C}^*), \dots , \mathrm{I}_8(\mathbf {N},\mathbf {N}_\perp , \mathbb {C}^*)) \, , \quad p = 1,\dots ,8 \, . \end{aligned}$$

Then, using (6), Eq. (3) leads to the condition

7 $$\begin{aligned}&s_4[\mathbb {A}\mathbb {C}^* - \mathbb {C}^*\mathbb {A}] + s_5[\mathbb {A}(\mathbb {C}^*)^2 - (\mathbb {C}^*)^2\mathbb {A}] + s_6[\mathbb {A}_\perp \mathbb {C}^* - \mathbb {C}^*\mathbb {A}_\perp ] + \\&\quad + s_7[\mathbb {A}_\perp (\mathbb {C}^*)^2 - (\mathbb {C}^*)^2\mathbb {A}_\perp ] + s_8[\mathbb {D}\mathbb {C}^*-\mathbb {C}^*\mathbb {D}] = \mathbb {O}. \end{aligned}$$

Multiplying both sides of the equation by $\mathbb {Q}^T$ on the left and by $\mathbb {Q}$ on the right, one obtains

8 $$\begin{aligned}&s_4[\mathbb {A}^*\mathbb {C}- \mathbb {C}\mathbb {A}^*] + s_5[\mathbb {A}^*\mathbb {C}^2 - \mathbb {C}^2\mathbb {A}^*] + s_6[\mathbb {A}_\perp ^* \mathbb {C}- \mathbb {C}\mathbb {A}_\perp ^*] + \\&\quad + s_7[\mathbb {A}_\perp ^*\mathbb {C}^2 - \mathbb {C}^2\mathbb {A}_\perp ^*] + s_8[\mathbb {D}^*\mathbb {C}-\mathbb {C}\mathbb {D}^*] = \mathbb {O}, \end{aligned}$$

in which we have set $\mathbb {A}^* := \mathbb {Q}^T \mathbb {A}\mathbb {Q}$ and $\mathbb {D}^* := \mathbb {Q}^T \mathbb {D}\mathbb {Q}$. Exploiting now the fact that $\mathbb {A}_\perp ~=~ \mathbb {I} - \mathbb {A}-\mathbf{k}\otimes \mathbf{k}$, where $\mathbf{k}=\mathbf {N}\times \mathbf {N}_\perp$ is the axis of rotation of $\mathbb {Q}$, we get

9 $$\begin{aligned} \mathbb {A}^*[(s_4 - s_6)\mathbb {C}+ (s_5-s_7)\mathbb {C}^2] - [(s_4 - s_6)\mathbb {C}+ (s_5-s_7)\mathbb {C}^2]\mathbb {A}^* +s_8(\mathbb {D}^*\mathbb {C}- \mathbb {C}\mathbb {D}^*) = \mathbb {O}. \end{aligned}$$

If we focus on the last term on the left-hand side, elementary tensor algebra allows to rewrite it as

$$\begin{aligned} \mathbb {D}^*\mathbb {C}- \mathbb {C}\mathbb {D}^* = {{\,\mathrm{Sym}\,}}(\mathbb {Q}^T\mathbf {N}\otimes \mathbb {Q}^T\mathbf {N}_\perp )\mathbb {C}- \mathbb {C}{{\,\mathrm{Sym}\,}}(\mathbb {Q}^T\mathbf {N}\otimes \mathbb {Q}^T\mathbf {N}_\perp ) = {{\,\mathrm{Sym}\,}}(\mathbb {R}) \mathbb {C}- \mathbb {C}{{\,\mathrm{Sym}\,}}(\mathbb {R}), \end{aligned}$$

with

10 $$\begin{aligned} \mathbb {R} := \mathbb {Q}^T\mathbf {N}\otimes \mathbb {Q}^T\mathbf {N}_\perp \, , \end{aligned}$$

while the operator ${{\,\mathrm{Sym}\,}}$ takes the symmetric part of its tensorial argument. A direct substitution into (9) leads to

$$\begin{aligned} \left[ (s_4-s_6)\mathbb {A}^* + s_8{{\,\mathrm{Sym}\,}}(\mathbb {R}) + (s_5-s_7)\mathbb {C}\right] \mathbb {C}- \mathbb {C}\left[ (s_4-s_6)\mathbb {A}^* + s_8{{\,\mathrm{Sym}\,}}(\mathbb {R}) + (s_5-s_7)\mathbb {C}\right] = \mathbb {O}, \end{aligned}$$

or equivalently

11 $$\begin{aligned} \widehat{\mathbb {A}}\mathbb {C}- \mathbb {C}\widehat{\mathbb {A}} = \mathbb {O}, \end{aligned}$$

where

12 $$\begin{aligned} \widehat{\mathbb {A}} := (s_4-s_6)\mathbb {A}^* + s_8{{\,\mathrm{Sym}\,}}(\mathbb {R}). \end{aligned}$$

It is straightforward to observe that the conclusion is not influenced by the classical first three invariants $\mathrm{I}_1, \mathrm{I}_2$ and $\mathrm{I}_3$, which represent the isotropic response of the material and do not change with rotations. Hence, since we are interested in the anisotropic behaviour of the system as a consequence of cell orientation, their dependence will be dropped in the following discussion, though one should remember that these terms might appear in any coefficient or as an additional term in the energy.

Equation (11) states that stationary points are identified by the rotations $\mathbb {Q}$ about the *z*-axis which make the tensors $\mathbb {C}$ and $\widehat{\mathbb {A}}$ defined in (12) commute. As pointed out in Vianello (1996), there are always at least two solutions, which can be easily identified in this case. In fact, if $\mathbb {Q}$ is such that $\mathbb {Q}\mathbf {N}$ is along one of the principal directions, then, on the one hand, $\mathbb {A}^* = \mathbb {Q}^T\mathbb {A}\mathbb {Q}$ is diagonal and therefore commutes with $\mathbb {C}$, which is diagonal by definition. On the other hand, we have that $\mathrm{I}_8(\mathbf {N},\mathbf {N}_\perp ,\mathbb {C}^*) = \mathrm{I}_8(\mathbb {Q}^T\mathbf {N},\mathbb {Q}^T\mathbf {N}_\perp ,\mathbb {C})$ which vanishes. Consequently, $s_8(\mathrm{I}_8)$ is null as well because it is an odd function of $\mathrm{I}_8$, because of the symmetry requirements on $\mathcal {U}$ which is assumed to be even in $\mathrm{I}_8$.

We conclude that configurations with cells oriented along the principal stretching direction or perpendicularly to it always correspond to stationary points of the elastic energy.

However, one might have further equilibria for other possible rotations satisfying (11), which will depend in general on the specific energy functional considered. In the following we will show that, for a large class of elastic energies, there might be two symmetric equilibria and we will study the stability of all configurations.

## Stability conditions for a quadratic-like energy

If we use the reference frame with the axes along the principal stretching directions, so that $\mathbb {C}$ is diagonal, it is convenient to identify the cell major axis through the angle $\theta$ it forms with the *x*-axis, so that $\mathbf {N}= (\cos \theta , \sin \theta , 0)$ and $\mathbf {N}_\perp = (-\sin \theta ,\cos \theta ,0)$. This angle is univocally associated to a rotation $\mathbb {Q}$ in the framework of the previous Section and in the following will be used as our main variable. With this choice, the invariants in (2) read

13 $$\begin{aligned} \mathrm{I}_4&= \lambda _x \cos ^2\theta +\lambda _y \sin ^2\theta = (\lambda _x- \lambda _y )\cos ^2\theta +\lambda _y\,, \\ \mathrm{I}_5&=   \lambda _x^2\cos ^2\theta +\lambda _y^2 \sin ^2\theta = (\lambda _x^2-\lambda _y^2)\cos ^2\theta +\lambda _y^2\,, \\ \mathrm{I}_6&=   \lambda _x \sin ^2\theta +\lambda _y \cos ^2\theta = \lambda _x -(\lambda _x -\lambda _y )\cos ^2\theta \,, \\ \mathrm{I}_7&=   \lambda _x^2\sin ^2\theta +\lambda _y^2 \cos ^2\theta = \lambda _x^2-(\lambda _x^2-\lambda _y^2)\cos ^2\theta \,, \\ \mathrm{I}_8&=   - (\lambda _x-\lambda _y)\sin \theta \cos \theta \, , \end{aligned}$$

and can be compactly rewritten as

$$\begin{aligned} \hat{\mathrm{I}}_i=a_i\cos ^2\theta +b_i\,,\quad \mathrm{for}\quad i=4,5,6,7 \end{aligned}$$

where

14 $$\begin{aligned} \begin{array}{ll} a_4=\lambda _x- \lambda _y\,, & b_4 = \lambda _y-1 \,,\\ a_5=\lambda _x^2- \lambda _y^2=a_4(\lambda _x+ \lambda _y)\,, & b_5 = \lambda _y^2-1=b_4(\lambda _y+1)\,,\\ a_6=-(\lambda _x- \lambda _y)=-a_4 \,, & b_6 = \lambda _x-1 \,,\\ a_7=-(\lambda _x^2- \lambda _y^2)=-a_5=-a_4(\lambda _x+ \lambda _y)\,, & b_7 = \lambda _x^2-1=b_6(\lambda _x+1)\,. \end{array} \end{aligned}$$

### The elastic energy

In order to investigate the existence of other stationary configurations in addition to the trivial ones, and to study their stability, we need now to specialize the elastic energy, trying to keep it as general as possible. We consider then the class of elastic energies that can be written as a homogeneous second-order polynomial in the variables $\hat{\mathrm{I}}_i:=\mathrm{I}_i-1$, for $i=4,5,6,7$, and $\mathrm{I}_8$, plus a term related to the isotropic response:

15 $$\begin{aligned} {\mathcal {U}}(\mathbf{I})=\dfrac{1}{2} \mathbf{I}\cdot {\mathbb K} \mathbf{I}+\mathcal{V}\,, \end{aligned}$$

where $\mathbf{I} := \left( \hat{\mathrm{I}}_4,\hat{\mathrm{I}}_5,\hat{\mathrm{I}}_6,\hat{\mathrm{I}}_7,\mathrm{I}_8\right)$ and ${\mathbb K}$ is the symmetric matrix of coefficients (that might possibly depend on the isotropic invariants), while $\mathcal{V}$ is the purely isotropic contribution that depends on $(\mathrm{I}_1,\mathrm{I}_2,\mathrm{I}_3)$.

We observe that the following analysis can be straightforwardly repeated for a Fung-type energy

16 $$\begin{aligned} {\mathcal {U}}_F=C\left[ \exp \left( \dfrac{\mathcal {U}}{{\mathcal {U}}_0}-1\right) -1\right] \,, \end{aligned}$$

that is often used in biomechanical applications (Fung 1967), giving rise to the same results. In fact, the stationary points of $\mathcal {U}_F$ coincide with the ones of $\mathcal {U}$. Moreover, their stability character may be identified by the second derivative of $\mathcal {U}$ and then is the same as well. Therefore, the results obtained for a quadratic energy also hold for an exponential-like energy, amplifying the validity of our conclusions.

We also remark that, in order to slightly reduce the terms influencing the stability analysis, we do not consider here possible isotropic-anisotropic couplings in the energy (15), that is, we exclude terms like $\mathrm{I}_h\hat{\mathrm{I}}_l$, where $h \in \{1,2,3\}$ and $l \in \{4,5,6,7,8\}$. This can be easily done and their role will be analysed in a future work.

For future convenience, it is useful to denote by $k_{ij}$ for $i,j=4,\ldots ,8$ the coefficients of the matrix ${\mathbb K}$ (*e.g.*, $k_{44}$ is the coefficient in the top left corner of the matrix). In particular, the coefficient $k_{44}$ is related to the stiffness along the polarization axis of the cell and $k_{66}$ to the one along the direction orthogonal to the cell major axis. Considering that stress fibers are mainly aligned to the polarization direction, coherently with Butler et al. (2002) we will assume that

$$\begin{aligned} k_{44}>k_{66}\,. \end{aligned}$$

We also point out that the coefficient $k_{88}$ is related to the response to shear, i.e. at the microscopic level to the resistance of changing angle among actin fibers, also involving the action of actin cross-linking proteins, such as filamin, Rho/Rac GTPases (Civelekoglu-Scholey et al. 2005; Wang et al. 2001) and Arp2/3 as well (Goley and Welch 2006; Rouiller et al. 2008) (see Fig. 1).

We finally observe that, because of the symmetry conditions related to a switching of the orientation of the axis, the coefficients $k_{i8}$ (and $k_{8i}$), $i=~4,5,6,7$ must vanish. In fact, they multiply the cross terms $\hat{\mathrm{I}}_i\mathrm{I}_8$ that change sign under these symmetry transformations. Then, their inclusion would break the symmetry of the energy, leading to a biologically unfeasible situation.

### Stationary configurations

Taking into account the consequences of symmetry on the coefficients of matrix ${\mathbb K}$ in (15), the stationary configurations are then identified by the solutions of

$$\begin{aligned} \mathbf{I}(\theta )\cdot {\mathbb K}\dfrac{\partial \mathbf{I}}{\partial \theta }(\theta )=0\,, \end{aligned}$$

that can be explicitly written as

$$\begin{aligned} \sum _{i,j=4}^7 k_{ij}\hat{\mathrm{I}}_i \dfrac{\partial \hat{\mathrm{I}}_j}{\partial \theta }+k_{88}\mathrm{I}_8\dfrac{\partial \mathrm{I}_8}{\partial \theta }=0\,. \end{aligned}$$

Recalling the form of the coefficients (14), the previous equation reads

$$\begin{aligned} \left[ -2\sum _{i,j=4}^7 k_{ij} a_i a_j \cos ^2\theta -2\sum _{i,j=4}^7 k_{ij} b_i a_j+k_{88} a_4^2(\cos ^2\theta -\sin ^2\theta )\right] \sin \theta \cos \theta =0\,. \end{aligned}$$

As already discussed at the end of the previous Section, the configurations with $\theta =0$ and $\theta = \pi /2$ (which in the following will sometimes be referred to as *parallel* and *perpendicular* orientation, respectively) are always stationary. However, there might be additional equilibria satisfying

$$\begin{aligned} \cos ^2\theta _{\mathrm{eq}}=\dfrac{1}{2a_4}\,\dfrac{2\Sigma _2 - k_{88}a_4}{\Sigma _1 - k_{88}}\,, \end{aligned}$$

or

17 $$\begin{aligned} \cos ^2\theta _{\mathrm{eq}}=\dfrac{1}{2}-\,\dfrac{1}{2}\,\dfrac{\Sigma _1}{\Sigma _1 - k_{88}}+\dfrac{1}{a_4}\,\dfrac{\Sigma _2}{\Sigma _1 - k_{88}}\,, \end{aligned}$$

where

$$\begin{aligned} \Sigma _1:=\dfrac{1}{a_4^2}\sum _{i,j=4}^7 k_{ij} a_i a_j\,,\qquad \mathrm{and}\qquad \Sigma _2:=-\,\dfrac{1}{a_4}\sum _{i,j=4}^7 k_{ij} b_i a_j\,, \end{aligned}$$

that can also be explicited as

$$\begin{aligned} \Sigma _1 =k_{44}-2k_{46}+k_{66}+ 2(k_{45}-k_{47}-k_{56}+k_{67})(\lambda _x+\lambda _y)+ (k_{55}-2k_{57}+k_{77})(\lambda _x+\lambda _y)^2, \end{aligned}$$

and

$$\begin{aligned} \Sigma _2&=   k_{44}(1-\lambda _y)+k_{55}(\lambda _x+\lambda _y)(1-\lambda _y^2)+ k_{66}(\lambda _x-1)+k_{77}(\lambda _x+\lambda _y)(\lambda _x^2-1)\\&+ k_{45}(1-\lambda _y)(\lambda _x+2\lambda _y+1)-k_{46}(\lambda _x-\lambda _y)- k_{47}(\lambda _x^2-\lambda _y^2-\lambda _x \lambda _y+ \lambda _x +\lambda _y -1)\\&- k_{56}(\lambda _x^2-\lambda _y^2+\lambda _x \lambda _y- \lambda _x -\lambda _y +1) - k_{57}(\lambda _x+\lambda _y)^2(\lambda _x- \lambda _y)+ k_{67}(\lambda _x-1)(2\lambda _x+\lambda _y+1). \end{aligned}$$

The nontrivial solution $\theta _{\mathrm{eq}}$, also called *oblique* orientation, exists if and only if

18 $$\begin{aligned} 0 \le \dfrac{2\Sigma _2 - k_{88}a_4}{\Sigma _1 - k_{88}} \le 2a_4\,, \end{aligned}$$

or equivalently if

19 $$\begin{aligned} \dfrac{2\Sigma _2 - k_{88}a_4}{\Sigma _1 - k_{88}} \ge 0\quad \mathrm{and}\quad \dfrac{2\Sigma _2 - 2 a_4 \Sigma _1 + k_{88}a_4}{\Sigma _1 - k_{88}} \le 0\,. \end{aligned}$$

### Stability

As far as stability is concerned, we need to evaluate the second derivative of the energy

$$\begin{aligned} \mathcal {U}''(\theta ) &=\Biggl [4 \sum _{i,j=4}^7 k_{ij}a_i a_j \cos ^2\theta \sin ^2\theta - 2\sum _{i,j=4}^7 k_{ij} a_j (a_i \cos ^2\theta + b_i)(\cos ^2\theta -\sin ^2\theta ) \\&+ k_{88} a_4^2 \left( (\cos ^2\theta -\sin ^2\theta )^2 - 4\cos ^2\theta \sin ^2\theta \right) \Biggr ]. \end{aligned}$$

Imposing the positivity of $\mathcal {U}''$ then leads to the following stability conditions:

20 $$\begin{aligned} \begin{array}{ccl} \theta =0: \quad &\mathrm{stable}\quad \Longleftrightarrow & 2\Sigma _2-2a_4\Sigma _1+k_{88}a_4>0\,,\\ \theta =\dfrac{\pi }{2}: \quad &\mathrm{stable}\quad \Longleftrightarrow & 2\Sigma _2-k_{88}a_4<0\,,\\ \theta =\theta _{\mathrm{eq}}: \quad &\mathrm{stable}\quad \Longleftrightarrow & 2\Sigma _2 - k_{88}a_4 \ge 0\quad \mathrm{and}\quad 2\Sigma _2 - 2 a_4 \Sigma _1 + k_{88}a_4 \le 0\,, \end{array} \end{aligned}$$

where in the last condition we have used the fact that $\mathcal {U}''(\theta _{\mathrm{eq}}) > 0$ requires $\Sigma _1-k_{88}>0$ to simplify the existence condition (19).

In the following, we will sometimes refer to the preferential orientations of Eq. (20) as *equilibrium angles*. However, we remark that they are not in general solutions of the elastic problem, but only stationary points of the elastic energy.

## Bifurcation results

Starting from the computations performed above, in this Section by means of a bifurcation analysis we discuss the preferential orientations of cells on a stretched substrate, for an elastic energy given by (15) or (16). We firstly discuss the case in which the strain energy does not depend on the invariants related to $\mathbb {C}^2$, and then we analyse the effects of their correction, since they introduce a significant change in the predicted behaviour. In particular, we consider three types of finite deformation: in the first one we fix the stretch in the *y*-direction $\lambda _y$ to a certain value and $\lambda _z = 1$, while letting $\lambda _x$ vary. In the second one we keep $\lambda _x \lambda _y = 1$, corresponding to an isochoric planar deformation if $\lambda _z = 1$. Finally, we set $\lambda _y = 1/\sqrt{\lambda _x}$, equivalent to a pure isochoric deformation that involves also the *z*-direction. Although we do not have any experimental information on $\lambda _z$, it does not affect the discussion on equilibria or on their stability character.

### Independence from $\mathbb {C}^2$

Let us firstly consider the case in which the elastic energy is independent from terms containing $\mathbb {C}^2$, that is, from the anisotropic invariants $\mathrm{I}_5$ and $\mathrm{I}_7$. This case can be easily obtained from the previous computations setting $k_{45}=k_{47} = k_{55} = k_{56} = k_{57} = k_{67} = k_{77}=0$. Doing so, we can get simplified forms for the terms

$$\begin{aligned} \Sigma _1=k_{44}-2k_{46}+k_{66}\,, \end{aligned}$$

and

$$\begin{aligned} \Sigma _2=k_{44}(1-\lambda _y)-k_{46}(\lambda _x-\lambda _y)+k_{66}(\lambda _x-1)\,. \end{aligned}$$

Hence, in addition to the trivial equilibria, Eq. (17) simplifies to

21 $$\begin{aligned} \cos ^2\theta _{\mathrm{eq}}=\dfrac{1}{2}+\dfrac{k_{44}-k_{66}}{k_{44}+k_{66}-2k_{46}-k_{88}} \left( \dfrac{1}{2}\,-\,\dfrac{\lambda _x-1}{\lambda _x-\lambda _y}\right) \,, \end{aligned}$$

putting in clear evidence a linear relationship between $\cos ^2\theta _{\mathrm{eq}}$ and the parameter

$$\begin{aligned} \Lambda :=\dfrac{\lambda _x-1}{\lambda _x-\lambda _y}\,, \end{aligned}$$

which compares the elongation along *x* with respect to the sum of elongation and contraction along *y*.

**Fig. 2:**
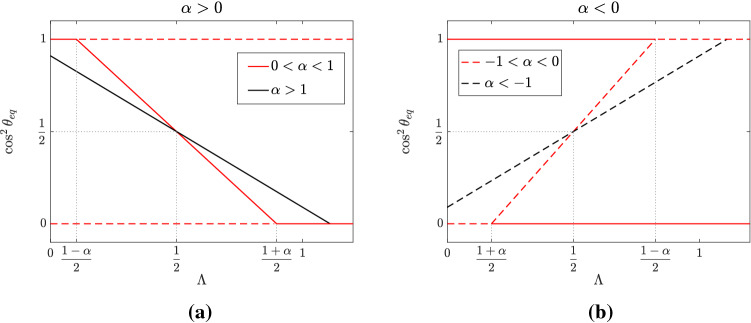
Bifurcation diagram of equilibrium angles in terms of $\Lambda :=\frac{\lambda _x-1}{\lambda _x-\lambda _y}$ with $\alpha :=~\frac{k_{44}+k_{66}-2k_{46}-k_{88}}{k_{44}-k_{66}}$ positive in **a** and negative in **b**. The black lines refer to the case $|\alpha |>1$ and the red lines to $|\alpha |<1$, while dashed lines represent unstable configurations and full lines stable ones. In (b) full lines indicating the stability of $\theta =0$ till the bifurcation point and of $\theta =\frac{\pi }{2}$ for $\alpha <-1$ are not drawn. The area with $\Lambda < 0$ is not shown because of the pulling characteristics of the experiments

The slope of the straight line (21) is determined by the inverse of the combination of coefficients

$$\begin{aligned} \alpha :=\frac{k_{44}+k_{66}-2k_{46}-k_{88}}{k_{44}-k_{66}}\,, \end{aligned}$$

as shown in Fig. 2. Actually, data from Faust et al. (2011), Livne et al. (2014) suggest a slope for the straight line in Eq. (21) of $-1.26\pm 0.08$, corresponding to $\alpha =0.794\pm 0.08$ (data reported in Fig. 5).

It is also worth to observe that the line always passes through the point corresponding to $\cos ^2\theta _{\mathrm{eq}} = 1/2$ (i.e. $\theta _{\mathrm{eq}} = \pi /4$) when $\Lambda = 1/2$, for any values of the elastic parameters. This is explained by the fact that, for a deformation satisfying $\Lambda = 1/2$ (or equivalently $\lambda _x - 1 = 1 - \lambda _y$), the minimum of the energy coincides with the direction of minimal strain. Indeed, the latter is given by the angle such that $\mathrm{I}_4 = 1$, that is,

$$\begin{aligned} \widetilde{\theta } = \arctan \left( \sqrt{\frac{\lambda _x - 1}{1-\lambda _y}}\right) = \arctan \left( \sqrt{\frac{\Lambda }{1-\Lambda }}\right) . \end{aligned}$$

Therefore, when $\Lambda = 1/2$, we have $\widetilde{\theta } = \pi /4$ which coincides with $\theta _{\mathrm{eq}}$ obtained using Eq. (21). This is not true in general, since $\widetilde{\theta }$ satisfies

$$\begin{aligned} \cos ^2\widetilde{\theta } = 1-\Lambda , \end{aligned}$$

differently from what is found through energy minimization in Eq. (21) except for the case $\alpha = 1$, which does not fit the experimental data (Faust et al. 2011; Livne et al. 2014). In fact, as pointed out in Livne et al. (2014), choosing the minimal strain direction as the preferential one for cell orientation does not allow to describe the experimental observations, while an energetic approach does. The only case in which the minimal strain direction and the minimal energy direction coincide is precisely when $\Lambda = 1/2$. These observations also allow us to justify even more the need of choosing an orthotropic constitutive model instead of a purely transversely isotropic one. In fact, the elastic strain energy for a transversely isotropic medium only depends on $\mathrm{I}_4$ and $\mathrm{I}_5$ in addition to the three isotropic invariants, and then only on $\mathrm{I}_4$ when the dependence on $\mathbb {C}^2$ is not considered as done in this Section. However, in such a case one obtains $\alpha =1$ and so minimizing a transversely isotropic energy would be equivalent to choosing the minimal strain direction again, which as just discussed does not seem accurate.

We also remark that $\Lambda =1$ corresponds to clamping the specimen, so that $\lambda _y=1$, while $\Lambda >1$ corresponds to stretching also along *y*, still keeping $\lambda _y<\lambda _x$. On the other hand, values of $\Lambda <\frac{1}{2}$ correspond to $\lambda _x-1<1-\lambda _y$, i.e. compressions along *y* are stronger than elongations along *x*, which is not done in experiments reported in the literature.

Finally, we observe the following cases related to isochoric deformations, which will be examined in detail later:

$$\begin{aligned} \begin{array}{ll} \mathrm{If}\quad \lambda _y=\dfrac{1}{\lambda _x}, \qquad &\mathrm{then}\quad \Lambda =\dfrac{\lambda _x}{1+\lambda _x}\in \left[ \dfrac{1}{2},1\right) ;\\ \mathrm{If}\quad \lambda _y=\dfrac{1}{\sqrt{\lambda _x}}, \qquad &\mathrm{then}\quad \Lambda =\dfrac{\lambda _x+\sqrt{\lambda _x}}{\lambda _x+\sqrt{\lambda _x}+1}\in \left[ \dfrac{2}{3},1\right) , \end{array} \end{aligned}$$

with $\Lambda \rightarrow 1$ for very large $\lambda _x$ and the lower extremum of the interval achieved in the limit of no stretching. Of course, for these types of finite deformation, the case $\Lambda > 1$ is unfeasible.

In terms of $\alpha$ and $\Lambda$ the existence condition for the non-trivial equilibrium (18) writes as

$$\begin{aligned} \dfrac{1-|\alpha |}{2}\le \Lambda \le \dfrac{1+|\alpha |}{2}\,. \end{aligned}$$

#### Case $\alpha > 0$

When $\alpha >0$, the stability conditions (20) become

22 $$\begin{aligned} \begin{array}{ccl} \theta =0: \quad \mathrm{stable}\quad & \Longleftrightarrow & \Lambda <\dfrac{1-\alpha }{2}\,,\\ \\ \theta =\dfrac{\pi }{2}: \quad \mathrm{stable}\quad & \Longleftrightarrow & \Lambda >\dfrac{1+\alpha }{2}\,,\\ \\ \theta =\theta _{\mathrm{eq}}: \quad \mathrm{stable}\quad & \Longleftrightarrow & \Lambda \in \left[ \dfrac{1-\alpha }{2},\dfrac{1+\alpha }{2}\right] , \end{array} \end{aligned}$$

that is, the non-trivial equilibrium position is stable whenever it exists.

Recalling that $k_{44}>k_{66}$ and focusing for the moment only on the case $\alpha >0$, there are two relevant subcases to discuss, depending on the following relationships between parameters:

(i) $$k_{46}+\dfrac{k_{88}}{2}<k_{66}\qquad \Longleftrightarrow \qquad \alpha >1 \, , \quad \text { or }$$
(ii) $$k_{66}<k_{46}+\dfrac{k_{88}}{2}<\dfrac{k_{44}+k_{66}}{2}\qquad \Longleftrightarrow \qquad 0<\alpha <1$$.

Referring to Fig. 2a, in case (ii)—represented by red lines—we have two supercritical bifurcation points at

$$\begin{aligned} \Lambda =\frac{1\pm \alpha }{2}. \end{aligned}$$

The configuration with orientation perpendicular to the stretching direction (i.e. $\theta _{\mathrm{eq}}=\pi /2$, $\cos ^2\theta _{\mathrm{eq}}=0$) is stable if $\Lambda >\frac{1+\alpha }{2}$. When $\Lambda$ is decreased below this value, the polarization axis of the cell tends instead to orient obliquely, finally becoming completely aligned to the stretching direction if $\Lambda <\frac{1-\alpha }{2}$. However, in case (i), represented by the black line in Fig. 2a, this value is negative and so it cannot be physically achieved in the usual experimental set-up. In this situation, cells will never orient themselves along the stretching direction.

In order to compare this behaviour with previous linear elasticity results, we observe that if we define $\varepsilon$ and *r* such that $\lambda _x=1+\varepsilon$ and $\lambda _y=1-r\varepsilon$, we have that $\Lambda =\frac{1}{1+r}$. So, one recovers the linear relationship between $\cos ^2\theta$ and $\frac{1}{1+r}$ discussed in Livne et al. (2014). Actually, since $\alpha =0.794\pm 0.08<1$, the situation observed in their experiments seems to correspond to case (ii). In this case, the two bifurcation points fall in the interval [0, 1]. We recall that $\Lambda =1$ corresponds to $\lambda _y=1$, that is no stretching in the *y*-direction, i.e. a clamped condition.

The analysis done here shows, in particular, that any model of type (15) or of Fung type (16) independent of $\mathrm{I}_5$ and $\mathrm{I}_7$ (i.e. the invariants depending on $\mathbb {C}^2$) with the following relation

23 $$\begin{aligned} \frac{k_{44}+k_{66}-2k_{46}-k_{88}}{k_{44}-k_{66}}\approx 0.794 \end{aligned}$$

among the coefficients is able to fit the parameters, in a way that is independent of the magnitude of the applied strain, even outside the range of validity of linear elasticity. This explains why the experimental behaviour shown in Faust et al. (2011), Livne et al. (2014) seems to be independent or nearly independent of the magnitude of the applied strain.

#### Case $\alpha < 0$

For the sake of completeness, we analyse the case in which $\alpha <0$, which can occur, for instance, if $k_{88}$ is much larger than the other parameters. In this case, noticing that $\frac{1+\alpha }{2} < \frac{1-\alpha }{2}$, the stability conditions are the following:

24 $$\begin{aligned} \begin{array}{ccl} \theta =0: \quad \mathrm{stable}\quad & \Longleftrightarrow & \Lambda <\dfrac{1-\alpha }{2}\,,\\ \\ \theta =\dfrac{\pi }{2}: \quad \mathrm{stable}\quad & \Longleftrightarrow & \Lambda>\dfrac{1+\alpha }{2}\,,\\ \\ \theta =\theta _{\mathrm{eq}}: \quad \mathrm{unstable} & \quad & \forall \Lambda > 0. \end{array} \end{aligned}$$

More precisely, also in this case there are two distinct situations to be considered:

(iii) $$\dfrac{k_{44}+k_{66}}{2}<k_{46}+\dfrac{k_{88}}{2}<k_{44}\qquad \Longleftrightarrow \qquad -1<\alpha <0 \, , \quad \text { or }$$
(iv) $$k_{44}<k_{46}+\dfrac{k_{88}}{2}\qquad \Longleftrightarrow \qquad \alpha <-1\,.$$

Referring to Fig. 2b, in case (iii)—corresponding to red lines—one has two admissible subcritical bifurcation points with coexistence of two critical equilibria if $\Lambda \in \left[ \frac{1+\alpha }{2},\frac{1-\alpha }{2}\right]$, corresponding to the parallel and perpendicular orientation, and only one of them stable outside this range. In case iv) the equilibrium $\theta =\pi /2$ is always stable while $\theta =0$ is stable only if $\Lambda <\frac{1-\alpha }{2}$. In all cases, if $\alpha < 0$ the oblique orientation is unstable.

### $\mathbb {C}^2-$correction

We now analyse the correction that will be introduced if a dependence of the energy on the invariants depending on $\mathbb {C}^2$ (i.e. $\mathrm{I}_5$ and $\mathrm{I}_7$) is allowed. For this purpose, it is convenient to rewrite

$$\begin{aligned} \Sigma _1 =k_{44}-2k_{46}+k_{66}+A_1\, , \end{aligned}$$

with

$$\begin{aligned} A_1:=2(k_{45}+k_{47}-k_{56}+k_{67})(\lambda _x+\lambda _y)+ (k_{55}-2k_{57}+k_{77})(\lambda _x+\lambda _y)^2\,, \end{aligned}$$

and

$$\begin{aligned} \Sigma _2 =(k_{44}-k_{46}+A_2)(\lambda _x-\lambda _y)-(k_{44}-k_{66}+B_2)(\lambda _x-1)\, , \end{aligned}$$

where

$$\begin{aligned} A_2 :&=[k_{55}(\lambda _y+1)-k_{47}-k_{56}-k_{57}(\lambda _x+\lambda _y)](\lambda _x+\lambda _y) +k_{45}(\lambda _x+2\lambda _y+1)-\dfrac{k_{47}-k_{56}}{2} (\lambda _x-1) \, ,\\ B_2 :&=[k_{55}(\lambda _y+1)-k_{77}(\lambda _x+1)](\lambda _x+\lambda _y) +k_{45}(\lambda _x+2\lambda _y+1) - k_{67}(2\lambda _x+\lambda _y+1)\\&- (k_{47}- k_{56})\left( \lambda _x -1-\dfrac{\lambda _x-\lambda _y}{2}\right) \,. \end{aligned}$$

After computation, we can explicit the nontrivial equilibrium (17) as

25 $$\begin{aligned} \cos ^2\theta _{\mathrm{eq}}&=   \dfrac{k_{44}-k_{46}-\frac{k_{88}}{2}+A_2-(k_{44}-k_{66}+B_2)\Lambda }{k_{44}+k_{66}-2k_{46}-k_{88}+A_1} \\&=   \dfrac{1}{2}+\dfrac{k_{44}-k_{66}}{k_{44}+k_{66}-2k_{46}-k_{88}+A_1}\left( \dfrac{1}{2}-\Lambda \right) -\dfrac{\frac{A_1}{2}-A_2+B_2\Lambda }{k_{44}+k_{66}-2k_{46}-k_{88}+A_1} \,. \end{aligned}$$

We notice that the terms $A_1$, $A_2$ and $B_2$ are the corrections related to the presence of $\mathbb {C}^2$-dependent terms through the invariants $\mathrm{I}_5$ and $\mathrm{I}_7$. Indeed, when the elastic energy does not include their contribution, we recover (21), i.e. the linear relationship between $\cos ^2\theta$ and $\Lambda$. However, since the correction coefficients depend on $\lambda _x$ and $\lambda _y$, Eq. (25) does not represent a straight line anymore. It is worth remarking that the last term in (25) represents a shift from the equilibrium angle $\theta =\pi /4$ that looks close to the one observed when $\Lambda =1/2$.

#### Small deformation limit

We observe that, in the limit of small deformation, the zero-th order approximations of the corrections become

$$\begin{aligned}&A_1 \approx \widetilde{A}_1 := 4(k_{45}+k_{55}-k_{47}-k_{56}+k_{67}-2k_{57}+k_{77})\, ,\\&A_2 \approx \widetilde{A}_2 := 2(2k_{45}+2k_{55}-k_{47}-k_{56}-2k_{57})\, ,\\&B_2 \approx \widetilde{B}_2 := 4(k_{45}+k_{55} - k_{67}-k_{77})\, , \end{aligned}$$

and in Eq. (25) the last term vanishes, so that the approximating curve is again a straight line:

26 $$\begin{aligned} \cos ^2\theta _{\mathrm{eq}}\approx \dfrac{1}{2} +\dfrac{k_{44}-k_{66}+\tilde{B}_2}{k_{44}+k_{66}-2k_{46}-k_{88}+\tilde{A}_1} \left( \dfrac{1}{2}-\Lambda \right) \end{aligned}$$

such that $\theta _{\mathrm{eq}}=\pi /4$ when $\Lambda =1/2$ (that corresponds to $\lambda _x-1\approx 1-\lambda _y\ll 1$). Hence, in the linear limit, the only difference from the case analysed in the previous Section is that more coefficients contribute to the identification of the slope, or viceversa, given some experimental data it is hard to distinguish which coefficients contribute to the slope of the line. This is not surprising: in the linear limit, for instance, the contribution to the energy of $\mathrm{I}_4$ is indistinguishable from that of $\mathrm{I}_5$, and the dependence on $\mathrm{I}_6$ merges with the one on $\mathrm{I}_7$.

In fact, Eq. (26) can be rewritten in the same form as (21) with the following formal substitutions:

27 $$\begin{aligned}&k_{44} \quad \longrightarrow \quad K_\Vert = k_{44} + 4k_{45} + 4k_{55} \, , \end{aligned}$$

28 $$\begin{aligned}&k_{66} \quad \longrightarrow \quad K_\perp = k_{66} + 4k_{67} + 4k_{77} \, , \end{aligned}$$

29 $$\begin{aligned}&k_{46} \quad \longrightarrow \quad K_{\Vert \perp } = k_{46} + 2k_{47} + 2k_{56} + 4k_{57} \, . \end{aligned}$$

**Fig. 3:**
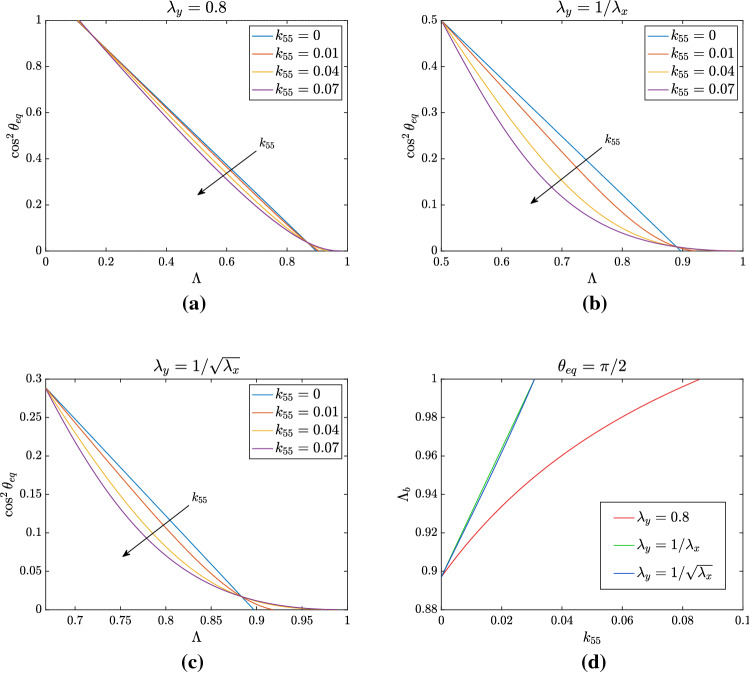
Analysis of non-trivial equilibrium position for $$k_{55} \ne 0$$ and $$k_{44}=0.4-4k_{55}$$ to keep the same linear limit. The other non-vanishing parameters are $$ k_{66}= k_{46} = 0.1$$, and $$k_{88}= 0.0618$$. In **a** $$\lambda _y=0.8$$ while in **b** $$\lambda _y=1/\lambda _x$$ and in **c** $$\lambda _y = 1/\sqrt{\lambda _x}$$. For all the three types of deformation, we see that the presence of $$k_{55}$$ leads to a departure from the linear relation between $$\cos ^2\theta _{\mathrm{eq}}$$ and $$\Lambda $$. In **d** the bifurcation point $$\Lambda _b$$ for which $$\theta _{\mathrm{eq}} = \pi /2$$ is shown as a function of $$k_{55}$$: the plot highlights that, for sufficiently high values of $$k_{55}$$ (for instance $$k_{55}> 0.085$$ for $$\lambda _y=0.8$$), the bifurcation point disappears, since values of $$\Lambda _b > 1$$ are not admissible for the deformations we consider

In the following, in order to evaluate the influence of $\mathbb {C}^2$ corrections, we change one parameter at a time while keeping the slope of the straight line in (26) constant, in order to start from the same linear dependence. For instance, when $k_{55}> 0$, then, recalling (27), $k_{44}$ is decreased accordingly, so that the value of $K_\Vert$ is mantained constant. In particular, with this idea in mind, we fix the coefficients $K_{\Vert } = 0.4\, , K_{\Vert \perp } = 0.1 \, , K_\perp = 0.1$ and $k_{88}= 0.0618$ in order to match the experimental fitting value reported in Eq. (23).

#### Effect of $k_{55}$

In Fig. 3 we focus on the effect of a non-vanishing $k_{55}$, keeping to zero the other coefficients involving the indices 5 and 7. As already stated above, to keep the same value of $K_\Vert$ and therefore the same linear limit given by (26), as we vary $k_{55}$ we accordingly change $k_{44} = K_\Vert - 4k_{55}$. To observe how the non-trivial equilibrium is changed, we focus on the three types of deformation defined at the beginning of Sect. 5. One can observe that in all cases changing $k_{55}$ leads to a departure of the equilibrium curve from a straight line, becoming convex. In addition, keeping $\lambda _y$ fixed and changing $\Lambda$ as in Fig. 3a, one can appreciate a decrease in the value of $\cos ^2\theta _{\mathrm{eq}}$, which means an increase in the equilibrium angle, for $\Lambda =1/2$.

On the other hand, when $\lambda _x\lambda _y=1$ as in Fig. 3b, $\theta _{\mathrm{eq}}$ stays fixed at $\pi /4$ if $\Lambda = 1/2$. In fact, in the latter case $A_1=A_2=B_2=4k_{55}$ and the numerator of the last term in (25) vanishes for $\Lambda =1/2$. This does not occur in the former case. At the other extreme, when $\Lambda \rightarrow 1$, then the denominator of the second term in (25) grows indefinitely, while the last term tends to 1/2. So, the curve given by (25) tends back to 0 when $\Lambda \rightarrow 1$ (not visible in the figure because the curve becomes negative).

Finally, in the third case $\lambda _y = \sqrt{\lambda _x}$ shown in Fig. 3c we have a minimal possible value of $\Lambda = 2/3$, corresponding to an equilibrium angle such that $\cos ^2\theta _{\mathrm{eq}} = 0.29$, i.e. $\theta _{\mathrm{eq}}\approx 57^\circ$, that is sometimes reported in some discussions of experiments (see, for instance, Wang 2000; Wang et al. 1995).

As regards the bifurcation point between the equilibrium branch $\theta =\pi /2$ and the one given by (25), it is implicitly defined by

30 $$\begin{aligned} \Lambda _b = \frac{k_{44}-k_{46}-\frac{k_{88}}{2}+A_2}{k_{44}-k_{66}+B_2}, \end{aligned}$$

since the coefficients $A_2$ and $B_2$ also depend on $\Lambda _b$. Therefore, while we have seen that the introduction of nonlinear terms depending on $\text {I}_4$, $\text {I}_6$ and $\text {I}_8$ does not modify the bifurcation obtained in the linearized theory, the dependence of the energy on the invariants related to $\mathbb {C}^2$ entails an identifiable shift of the bifurcation point between the perpendicular orientation and the oblique one that may also disappear for large values of $k_{55}$, as shown in Fig. 3d.

If we assume $k_{55} \ne 0$, the shift of the bifurcation point for $\theta _{\mathrm{eq}} = \pi /2$ can be evaluated through

31 $$\begin{aligned} \Lambda _b=\dfrac{k_{44}-k_{46}-\frac{k_{88}}{2}+k_{55}(1+\lambda _y)(\lambda _x+\lambda _y)}{k_{44}-k_{66}+k_{55}(1+\lambda _y)(\lambda _x+\lambda _y)}\,. \end{aligned}$$

This means that, for given coefficients but $k_{55}$, (31) implicitly defines $\Lambda _b$ in terms of $k_{55}$ that, actually, can be made explicit by writing

32 $$\begin{aligned} k_{55}=\dfrac{(k_{44}-k_{66})\Lambda _b-k_{44}+k_{46}+\frac{k_{88}}{2}}{(1-\Lambda _b)(1+\lambda _y)(\lambda _x+\lambda _y)}\,. \end{aligned}$$

We notice that, in the equation above, the term $(1+\lambda _y)(\lambda _x+\lambda _y)$ is a function of $\Lambda _b$, given, for instance, by

33 $$\begin{aligned} (1+\lambda _y)(\lambda _x+\lambda _y) = \frac{1-2\Lambda _b+2\Lambda _b^2}{\Lambda _b^2(1-\Lambda _b)} \qquad \text { if } \lambda _x\lambda _y=1 \, , \end{aligned}$$

and by

34 $$\begin{aligned} (1+\lambda _y)(\lambda _x+\lambda _y) = (1+\bar{\lambda }_y)\frac{1+\bar{\lambda }_y-2\bar{\lambda }_y\Lambda _b}{1-\Lambda _b} \qquad \text { for fixed } \lambda _y=\bar{\lambda }_y. \end{aligned}$$

If $\lambda _y = 1/\sqrt{\lambda _x}$ we have that

$$\begin{aligned} 1-\Lambda _b = \frac{1}{\lambda _x + \sqrt{\lambda _x} + 1} \end{aligned}$$

which corresponds to

$$\begin{aligned} \lambda _x = \left( -\frac{1}{2}+\frac{1}{2}\sqrt{\frac{4}{1-\Lambda _b}-3}\,\right) ^2. \end{aligned}$$

These relations can be exploited to plot the variation of the bifurcation point as a function of $k_{55}$, as shown in Fig. 3d. The bifurcation point $\Lambda _b$ approaches 1 for increasing values of $k_{55}$, eventually leading to a disappearance of the bifurcation for some critical values of the parameter. To be more specific, if $\lambda _y=0.8$, for $k_{55}> 0.085$ the branch relative to the oblique equilibrium and $\theta =\pi /2$ do not cross for physically admissible values of $\Lambda$ and therefore the perpendicular orientation is always unstable. In particular, we observe that in the case of fixed $\lambda _y$ the threshold value is higher, while for the other two types of deformation it is the same and amounts to $k_{55}=k_{88}/2$. This follows immediately from (32) and (33) for $\lambda _y = 1/\lambda _x$, while in the case $\lambda _y = 1/\sqrt{\lambda _x}$ it is enough to observe that

35 $$\begin{aligned} (1-\Lambda _b)(1+\lambda _y)(\lambda _x+\lambda _y) = \left( 1-\Lambda _b\right) \left( 1+\frac{1}{\sqrt{\lambda _x}}\right) \left( \lambda _x + \frac{1}{\sqrt{\lambda _x}}\right) = 1 + \frac{1}{\lambda _x} - 2\left( 1-\Lambda _b\right) \,. \end{aligned}$$

Substituting into (32) and recalling that $\Lambda _b \rightarrow 1$ means $\lambda _x \rightarrow + \infty$ immediately leads to $k_{55} =~k_{88}/2$, coherently with Fig. 3d.

**Fig. 4:**
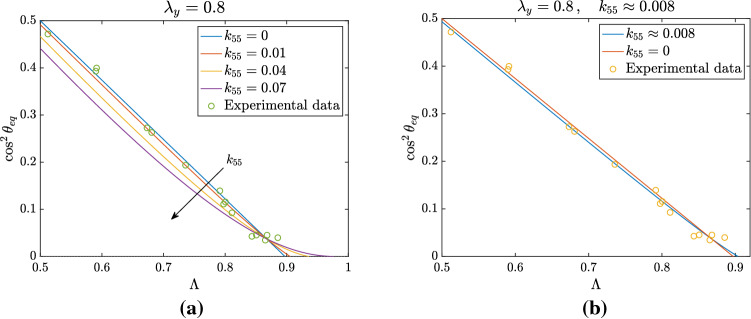
Nonlinear fitting of experimental data with $k_{55} \ne 0$. Data from Livne et al. (2014) are compared with the family of curves obtained for $\lambda _y = 0.8$ and different values of $k_{55}$ in **a** and with the best fitting value $k_{55} = 0.008$ in **b**

**Fig. 5:**
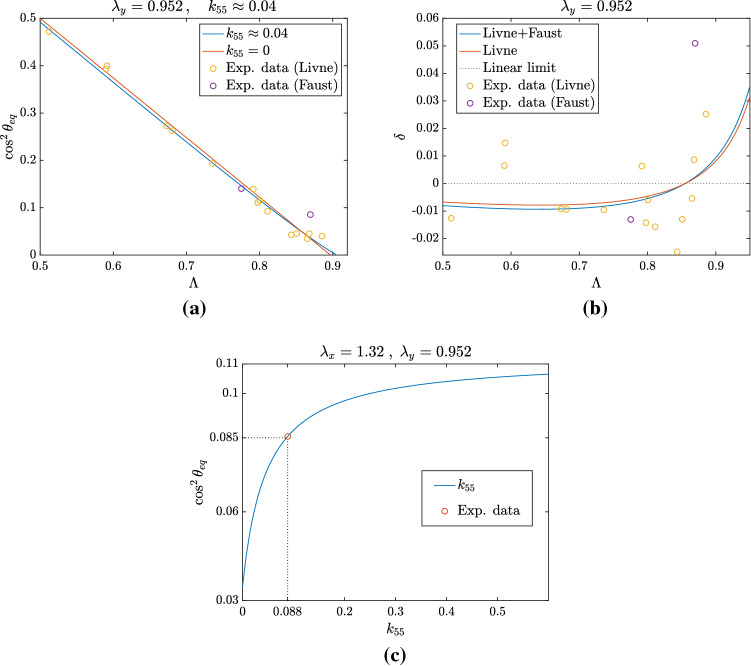
Nonlinear fitting of experimental data from Faust et al. (2011) and Livne et al. (2014). **a** Best fitting for $\lambda _y =~0.952$ obtained for $k_{55} \approx 0.04$. **b** Deviation $\delta$ from the straight line when fitting all data (blue curve) and only data from Livne et al. (2014) for a fixed $\lambda _y =~0.952$ (red curve; in this case, the best $k_{55} \approx 0.03$). **c** Equilibrium orientation as a function of $k_{55}$ for $\lambda _x=1.32$ and $\lambda _y=0.952$, related to experimental actin orientations obtained by Faust et al. (2011) for a 32% stretch (represented by the circle). As shown, a value of $k_{55} = 0.008$ is able to capture the experimental orientation precisely

In order to evaluate the relevance of the nonlinear correction, we performed a fitting of experimental data extracted from Livne et al. (2014) where, using a biaxial experiment, the stretches in the two directions are actually controlled. Although we do not know the exact value of $\lambda _y$, we observe that if $\lambda _y=1$, then $\Lambda$ would be identically equal to 1. So, we fix it to be $\lambda _y = 0.8$. Then, focusing on Fig. 4, we explored the possibility of a better fitting using nonlinear elasticity and $\mathbb {C}^2$ correction. Actually, as already observed, data are already fitted quite well by the straight line. However, using a nonlinear regression estimation we find that a small value of $k_{55} = 0.008$ gives an even better fitting for data in Livne et al. (2014). We also find that, if we increase the fixed value of $\lambda _y$, a higher value of the coefficient $k_{55}$ is needed to fit the data. For instance, when we take $\lambda _y = 0.98$ we find a best fitting value of $k_{55} = 0.08$, an order of magnitude greater than the one obtained for $\lambda _y = 0.8$. This is due to the fact that, when $\lambda _y \approx 1$, a small stretch $\lambda _x$ in the *x*-direction is sufficient to span all the admissible values of $\Lambda$. Consequently, nonlinear effects become less relevant and to fit the nonlinear model we need to take very high values of the related coefficients.

In Fig. 5, we present the fitting using also the data by Faust et al. (2011): we explored the fixed $\lambda _y$ case since, even if the Poisson ratio of the specimen is taken to be $\nu = 1/2$, the actual reported biaxiality ratio is different. In particular, considering the experimental settings with 32% and 31.7% strain, one finds a value of $\lambda _y = 0.952$ and $\lambda _y = 0.91$, respectively, since the biaxiality ratio is $\Lambda = 0.87$ and $\Lambda = 0.78$ in the two cases. In Fig. 5a, we then tried to fit the data (Faust et al. 2011; Livne et al. 2014) simultaneously to assess if a nonlinear correction could better explain the experimental observations in different settings. It is found that, for a fixed value of $\lambda _y = 0.952$, taking $k_{55} = 0.04$ gives a slightly better fitting than the straight line approximation, while nonlinear regression performed only on the data in Livne et al. (2014) returns a value of $k_{55} = 0.03$. To highlight this difference, in Fig. 5b we plot the deviation $\delta$ from the linear approximation, that is

36 $$\begin{aligned} \delta (\Lambda ) := \cos ^2\theta _{\mathrm{eq}}(\Lambda ) - \left[ \frac{1}{2}+\frac{k_{44}-k_{66}}{k_{44}+k_{66}-2k_{46}-k_{88}}\left( \frac{1}{2}-\Lambda \right) \right] , \end{aligned}$$

as a function of $\Lambda$. Since most of the experimental data fall below the straight line, i.e. below 0 in Fig. 5b, a convex curve obtained with the introduction of $k_{55}$ is able to better approximate the observed behaviour, even if the difference is of the order of $10^{-2}$.

Finally, we chose one of the experimental actin angle values obtained in Faust et al. (2011) for a 32% stretch and fixed $\lambda _x$ and $\lambda _y$ in order to have the same value of $\Lambda$ used in the experiment. Doing so, we tried to find a value of $k_{55}$ able to capture this experimental point: as shown in Fig. 5c, a value of $k_{55} = 0.088$ precisely fits the orientation angle for such a fixed deformation.

**Fig. 6:**
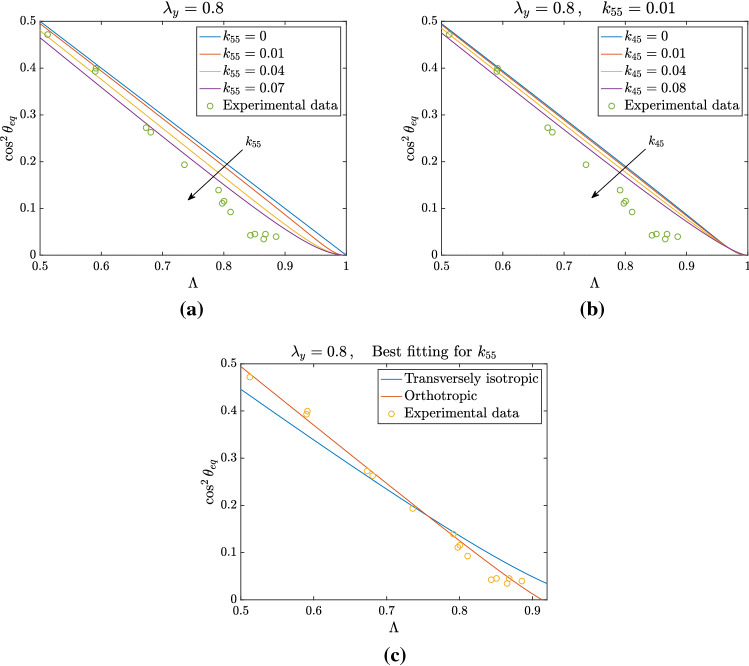
Nonlinear fitting of experimental data from Livne et al. (2014) with a transversely isotropic model, in the case $\lambda _y = 0.8$. In **a** the family of curves obtained from Eq. (37) for different values of $k_{55}$ is shown, while in **b** the effect of $k_{45}$ is investigated. In **c** a direct comparison between the best fitting curves for the transversely isotropic and orthotropic case is provided, showing that the latter is more accurate

We conclude our analysis by making a comparison with the transversely isotropic case, which we already discussed as inadequate to fit the experimental data. This is confirmed by the curves reported in Fig. 6. Indeed, if we consider a transversely isotropic energy that depends only on five invariants, recalling (25) we get:

37 $$\begin{aligned} \cos ^2\theta _{\mathrm{eq}} = \frac{k_{44}+k_{55}(\lambda _y+1)(\lambda _x+\lambda _y)+k_{45}(\lambda _x+2\lambda _y+1)}{k_{44}+2k_{45}(\lambda _x+\lambda _y)+k_{55}(\lambda _x+\lambda _y)^2}(1-\Lambda ). \end{aligned}$$

In Fig. 6a we plot the relationship (37) for different values of $k_{55}$ and $k_{45} = 0$, while in Fig. 6b the case $k_{45} \ne 0$ is shown. It is clearly observed that, in both cases, the transversely isotropic model provides a fitting which is not satisfactory compared to the orthotropic one reported in Fig. 4. To have a better insight, in Fig. 6c we show a direct comparison between the best fitting curves in the transversely isotropic and orthotropic case: in the latter, there is a significant improvement in the fitting of experimental data.

**Fig. 7:**
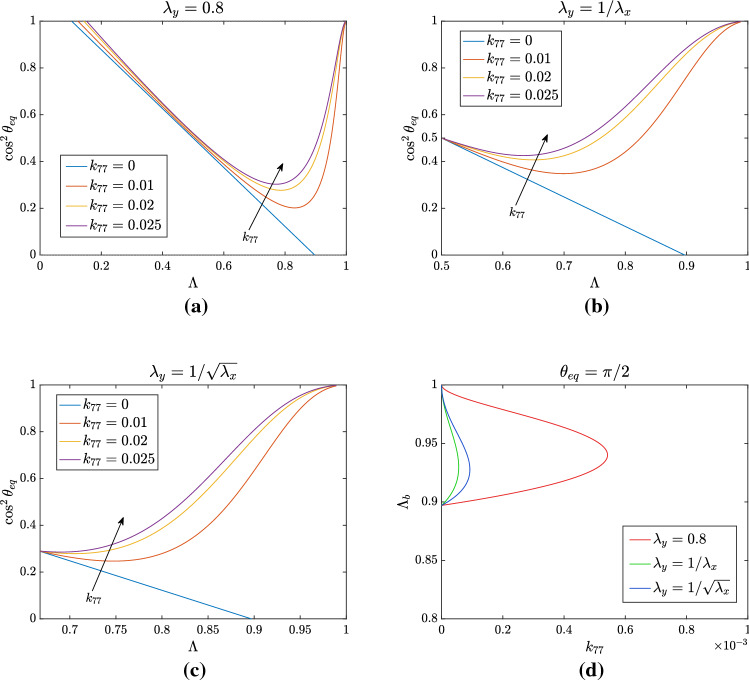
Analysis of non-trivial equilibrium position for $k_{77} \ne 0$ and $k_{66}~=~0.1-4k_{77}$ to keep the same linear limit. The other non-vanishing parameters are $k_{44}=0.4$, $k_{46} = 0.1$, and $k_{88}= 0.0618$. In **a** $\lambda _y=0.8$, while in **b** $\lambda _y=1/\lambda _x$ and in **c** $\lambda _y = 1/\sqrt{\lambda _x}$. For all the three types of deformation, one can see that introducing $k_{77}$ provokes a significant difference from the linear behaviour not observed in experiments, suggesting that such a parameter is not present in the constitutive model. In **d** the bifurcation points $\Lambda _b$ for which $\theta _{\mathrm{eq}} = \pi /2$ are shown as a function of $k_{77}$. The region enclosed by the curve identifies the values of $\Lambda$ for which $\theta _{\mathrm{eq}}=\pi /2$ is stable. For sufficiently high values of $k_{77}$ the bifurcation points disappear and the configuration $\theta _{\mathrm{eq}}=\pi /2$ is always unstable

#### Effect of $k_{77}$

We turn now the attention to the effect of a non-vanishing $k_{77}$, keeping $K_\perp$ fixed, and perform a similar reasoning as we did for $k_{55}$. Again, as shown in Fig. 7b, if $\lambda _x\lambda _y=1$ then $\theta =\pi /4$ when $\Lambda = 1/2$ for any value of $k_{77}$. In fact, in this case, $A_1=4k_{77}$ and $B_2=-4k_{77}$, while $A_2=0$. So, the numerator in the last term of (25) vanishes. This does not occur if $\lambda _y$ is kept fixed, as shown in Fig. 7a. In fact, the value of $\theta _{\mathrm{eq}}$ slightly decreases for increasing values of $k_{77}$.

A more dramatic effect occurs if $k_{77}\ne 0$ when $\Lambda \rightarrow 1$, because as before the second term in (25) tends to zero, but on the contrary of the previous case the last term tends to $-1/2$. This implies that the curve in (25) tends to 1 when $\Lambda \rightarrow 1$ and, as a consequence, there are two bifurcation values for the equilibrium configuration $\theta _{\mathrm{eq}} = \pi /2$ that is stable for values of $\Lambda$ in between them. However, the two bifurcation points soon disappear. Specifically, as shown in Fig. 7d, this happens for $k_{77}> 5.4\cdot 10^{-4}$, if $\lambda _y=0.8$ fixed. For such values, the equilibrium $\theta _{\mathrm{eq}}=\pi /2$ is always unstable, while the branch represented in Fig. 7 is always stable. This does not seem to correspond to what is observed in experiments, suggesting that $k_{77}=0$ or that it must have a very small value in the constitutive model.

**Fig. 8:**
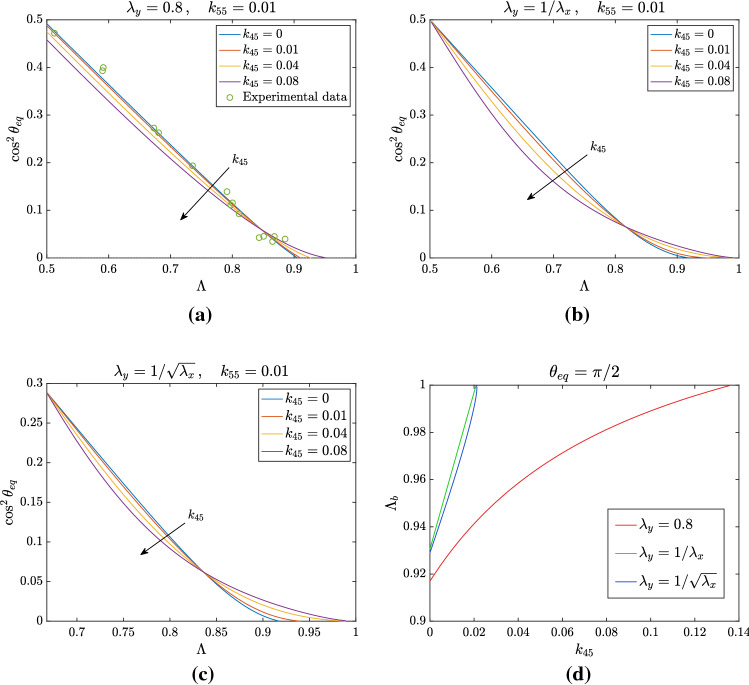
Analysis of nontrivial equilibrium position for $k_{45}\ne 0$, considering $k_{55} = 0.1$ and $k_{44} =~0.4-4k_{55}-4k_{45}$ to keep the same linear limit. The other non-vanishing parameters are $k_{46} = 0.1$ and $k_{88}= 0.0618$. In **a** we set $\lambda _y = 0.8$, while in **b** $\lambda _y = 1/\lambda _x$ and in **c** $\lambda _y = 1/\sqrt{\lambda _x}$. Moreover, in **a** we reported the experimental data obtained in Livne et al. (2014): the introduction of the mixing parameter $k_{45}$ does not improve significantly the fitting of such data. In (d) the bifurcation point $\Lambda _b$ for which $\theta _{\mathrm{eq}} = \pi /2$ is shown as a function of $k_{45}$, when $k_{55} = 0.01$ is kept fixed. For all the types of deformation considered, the bifurcation disappears when $k_{45}$ crosses a critical value, which is the same for $\lambda _y = 1/\lambda _x$ and $\lambda _y = 1/\sqrt{\lambda _x}$

#### Effect of $k_{45}$

Finally, we focus on the effect of coupling terms other than $k_{46}$, that is already present in the analysis of the previous Section, as it involves the right Cauchy–Green tensor $\mathbb {C}$. Having ruled out the importance of a non-vanishing $k_{77}$ we do not report the plots involving $k_{47}$, $k_{57}$ and $k_{67}$ and focus instead on the remaining mixing parameter $k_{45}$. The results of its introduction in the model are shown in Fig. 8. In particular, we fixed a value of $k_{55}=0.01$ and plotted the equilibrium angle as a function of $\Lambda$ for the three different deformations mentioned above. We observe an effect on the equilibrium curve similar to the one encountered increasing $k_{55}$. The departure from the straight line is slighter if $\lambda _y=0.8$, and greater in the other two cases. Focusing on Fig. 8d, as in the case $k_{55}\ne 0$, the bifurcation value above which the configuration $\theta _{\mathrm{eq}}=\pi /2$ is stable increases with $k_{45}$ and disappears for higher values of this parameter because the curve corresponding to the non-trivial configuration decreases to 1 (without a minimum).

As a last observation, comparing the theoretical results with experimental data shown in Fig. 8a it does not seem that the introduction of the mixing term $k_{45}$ is relevant for a better fit, as confirmed by nonlinear regression.

## Discussion

Stimulated by the need of understanding why the preferential configurations achieved by an ensemble of cells over a substratum under stretch were apparently independent of the mechanical characteristic of the substratum itself, of the stretch magnitude, and to a certain extent of the cell type, we investigated the phenomenon from a mechanical point of view. We then worked in the framework of nonlinear elasticity with a very general class of constitutive equations, often used in biomechanical applications, to describe the cell and the stretched substrate as an orthotropic material undergoing finite deformations.

Coherently with experiments and previous theoretical works that use linear elasticity, our model predicts that strained cells will reorient until their major axis forms a well-defined angle with the direction of stretching, depending on the biaxiality of the deformation quantified by the parameter $\Lambda$. We found that the bifurcation diagram only depends on a particular combination of the coefficients $k_{44}$, $k_{66}$, $k_{88}$ (in addition to the mixed one $k_{46}$) appearing in front of the terms with the anisotropic invariants $\mathrm{I}_4$, $\mathrm{I}_6$, and $\mathrm{I}_8$, that is, the ones determining the influence of $\mathbf {N}\cdot \mathbb {C}\mathbf {N}$, $\mathbf {N}_\perp \cdot \mathbb {C}\mathbf {N}_\perp$, and $\mathbf {N}_\perp \cdot \mathbb {C}\mathbf {N}$, where $\mathbf {N}$ is the direction of cell polarization and $\mathbf {N}_\perp$ the perpendicular one.

Hence, the results obtained using linear elasticity extend their validity also in the nonlinear regime and for a wide class of orthotropic models, which explains why experimental results with a stretch amplitude outside the linear elastic regime can be still well described by the linearized theory. In particular, our predictions confirm that there exists a linear relationship between $\Lambda$ and $\cos ^2\theta _{\mathrm{eq}}$, where $\theta _{\mathrm{eq}}$ is the angle formed by the aligned stress fibers of the cell and the main stretch direction, with slope that from experiments is given by

38 $$\begin{aligned} \frac{k_{44}-k_{66}}{k_{44}+k_{66}-2k_{46}-k_{88}}\approx 1.26\,. \end{aligned}$$

Therefore, we showed that any quadratic-like or Fung-type constitutive model independent of the invariants related to $\mathbb {C}^2$ (i.e. $\mathrm{I}_5$ and $\mathrm{I}_7$) can fit the experimental data, provided that the coefficients satisfy the relation (38). The only difference from the theoretical point of view is that, using finite elasticity, one has more coefficients contributing to the slope of the bifurcation line. Besides, our analysis is in principle able to describe the final reorientation angle even in the cases $\alpha < 0$ and $\alpha > 1$, which might be of interest in other experimental settings.

We also proved that for $\Lambda >\frac{1+\alpha }{2}\approx 0.897$ the only stable equilibrium is the one in which cells are aligned perpendicularly to the main stretching direction.

Then, we introduced the corrections due to the invariants related to $\mathbb {C}^2$ and analysed their influence. It is found that the presence of the invariant $\mathrm{I}_5$ involving $\mathbf {N}\cdot \mathbb {C}^2\mathbf {N}$ has an effect which is still compatible with experimental data, for small values of the pertaining coefficient $k_{55}$ and for all the three types of deformation considered. In this case, however, the dependence discussed above is no longer linear and the effect due to finite elasticity comes out clearly. This allows us to speculate that there might be a slight further nonlinear impact in the phenomenon, since the introduction of $k_{55}$ provided a slightly better fit to experimental data. The presence of such a parameter also influences the existence of a bifurcation between the perpendicular and the oblique orientation, which disappears for sufficiently high values of $k_{55}$. However, it is worth to remark that the data which are present in the literature seem very different: there is then a degree of uncertainty which does not allow to precisely conclude on the relevance of such a dependence. More experiments would be needed to assess the exact influence of this nonlinear parameter, progressively varying the stretching amplitude. From our results, it seems that performing experiments at values of $\Lambda$ close to one, i.e. nearly clamped experiments in the *y*-direction, would better put in evidence the effects of $\mathrm{I}_5$, as well as focusing on the moment when cells start departing from a perpendicular orientation with respect to the stretching direction, i.e. the bifurcation point occurring for high values of $\Lambda$.

Instead, it is found that a dependence of the strain energy on the invariant $\mathrm{I}_7$ involving $\mathbf {N}_\perp \cdot ~\mathbb {C}^2\mathbf {N}_\perp$ yields results that look incompatible with experiments. In particular, we showed that the presence of the coefficient $k_{77}$ leads to a consistent difference from the linear behaviour especially for high values of $\Lambda$ and to the disappearance of the bifurcation between the perpendicular and oblique orientation. Since these predictions are not confirmed by experimental data, we speculate that the constitutive model should not include its contribution. Finally, we showed that the influence of a mixing parameter related to $\mathbb {C}^2$ like $k_{45}$, which couples the effect of the invariants $\mathrm{I}_4$ and $\mathrm{I}_5$, is negligible and does not provide a better fitting to experimental data.

An aspect that is not covered by the present modelling is the dependence of the behaviour on the frequency of the applied cyclic stretch. In order to include that, one should take into account the viscoelastic properties of the material and in particular the characteristic response times of the cells with respect to the period of imposed oscillations. Some works in this direction have been done in Kong et al. (2008), Qian et al. (2013), who considered the viscoelasticity of actin stress fibers, mostly from a microscopic viewpoint, while in an ongoing work we are considering linear viscoelasticity regarding the system as a continuum.

More importantly, during the experimental tests there are remodelling effects related to the internal reorganization of the cytoskeleton and of the stress fibers. Introducing remodelling into the continuum structure of the model as done in Ciambella and Nardinocchi (2019), Ciambella and Nardinocchi (2020) could allow to explore this effect, maybe improving the description of such a complex behaviour of the cells.
